# Immunosensor of Nitrofuran Antibiotics and Their Metabolites in Animal-Derived Foods: A Review

**DOI:** 10.3389/fchem.2022.813666

**Published:** 2022-06-01

**Authors:** Jingze Jia, Hongxia Zhang, Jiayi Qu, Yuanfeng Wang, Naifeng Xu

**Affiliations:** Institute of Engineering Food, College of Life Science, Shanghai Normal Uniersity, Shanghai, China

**Keywords:** immunosensor, antibody, animal-derived foods, nitrofuran, antibiotics

## Abstract

Nitrofuran antibiotics have been widely used in the prevention and treatment of animal diseases due to the bactericidal effect. However, the residual and accumulation of their metabolites *in vivo* can pose serious health hazards to both humans and animals. Although their usage in feeding and process of food-derived animals have been banned in many countries, their metabolic residues are still frequently detected in materials and products of animal-derived food. Many sensitive and effective detection methods have been developed to deal with the problem. In this work, we summarized various immunological methods for the detection of four nitrofuran metabolites based on different types of detection principles and signal molecules. Furthermore, the development trend of detection technology in animal-derived food is prospected.

## 1 Introduction

Animal-derived foods, including livestock and poultry meat, eggs, aquatic products, milk and dairy products, are the main source for people to intake high-quality proteins, essential amino acids, minerals, vitamins and biologically active peptides. (Berner et al., 1985; Sampels, 2014). Over the past few decades, domestic animals have been infected by bacteria in the process of animal growth and breeding, due to factors such as non-standard environmental sanitation on farms and insufficient professional ability of farmers. Nitrofuran antibiotics can kill or inhibit germs, including Gram-positive bacteria, negative bacteria, pathogens, and are often used to treat animal diseases such as urinary and intestinal bacterial infections (Pontes and Groisman, 2020), or preventing diseases by being added to animal feeds.

**FIGURE 1:**
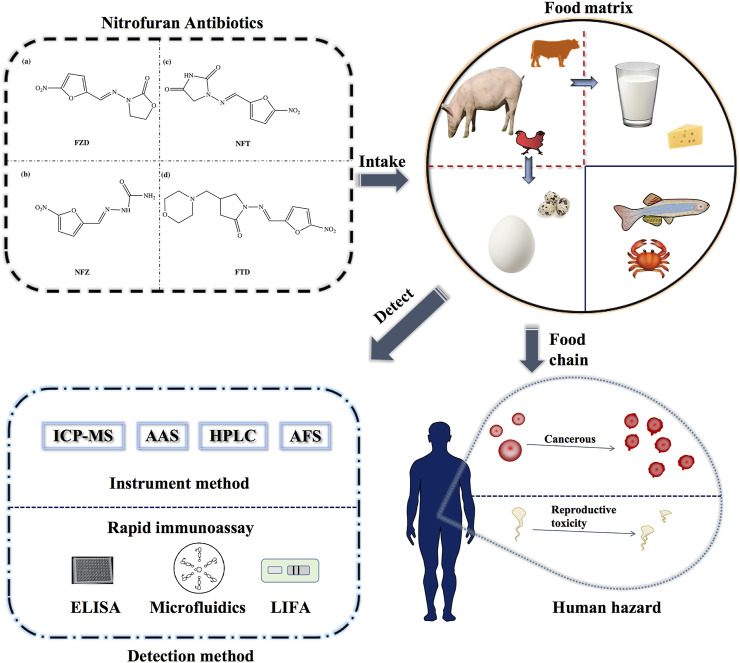
Schematic diagram of the detection methods of nitrofurans and the delivery process in the food chain.

Nitrofurans are a class of broad-spectrum antibiotics, mainly including furazolidone (FZD), nitrofurazone (NFZ), furaltadone (FTD), and nitrofurantoin (NFT). The structures are shown in Figure 1. They are used for promoting animal growth, treating poultry, and are very effective against gastrointestinal tract diseases and skin diseases of aquatic animals (Yu et al., 2013; Bacanli and Başaran, 2019). Due to the good effectiveness and low-cost investment there are often cases of excessive or illegal addition of these antibiotics. After acting on animals, such drugs can enter the bacterial cytoplasm, interfere with bacterial protein synthesis and sugar metabolism, and play a role in the antibacterial therapy (Calvo and Martínez-Martínez, 2009). Although the parent drugs metabolize rapidly *in vivo*, the metabolites which bind to tissue proteins in the form of complexes cannot be further metabolized, resulting in a large amount of metabolites remaining in the body. Studies have shown that the metabolic complexes accumulated in the body can induce cell carcinogenesis and affect animal health (Gong et al., 2020; Melekhin et al., 2021). The metabolites in animal body are relatively stable during storage and conventional cooking (Cooper and Kennedy, 2007). Common food processing methods (including grilling, microwave processing, cooking) are difficult to degrade the protein-bound metabolites, which are harmful to human healthy, in large quantities (Kennedy et al., 2000). Therefore, strict monitoring and detection of nitrofuran drugs and their metabolites residues in food are required.

**FIGURE 2:**
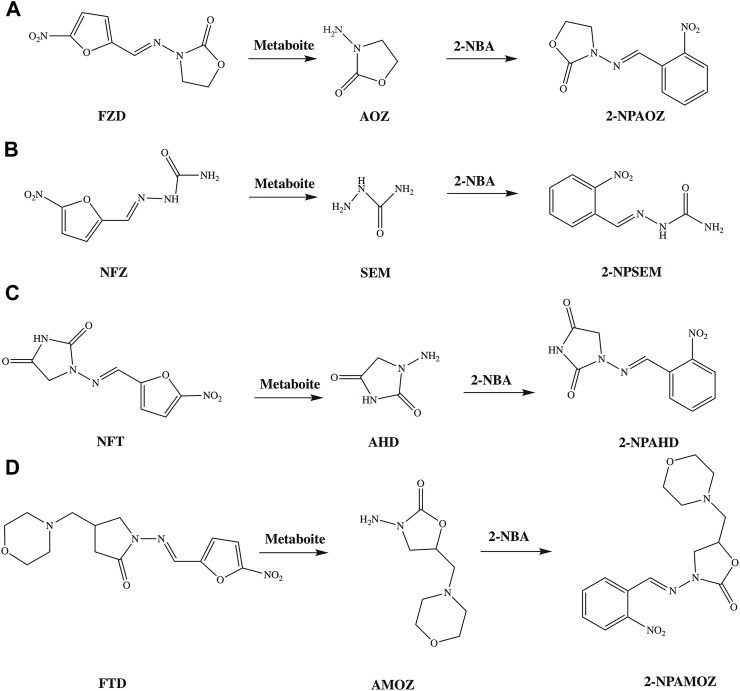
The structures of nitrofurans, their metabolites and commonly used derivatives. **(A)** The metabolism and derivation process of FZD; **(B)** metabolism and derivation process of NFZ; **(C)** metabolism and derivation process of NFT; **(D)** metabolism and derivation process of FTD.

Compared to the parent drugs, the metabolites are often employed as markers for the detection of nitrofuran antibiotics because of their long-term stability. The detection markers of furazolidone, nitrofurazone, furaltadone, and nitrofurantoin are 3-amino-2-oxazolidinone (AOZ), semicarbazide (SEM), 3-amino-5-morpholinomethyl-1, 3-oxazolidinon (AMOZ) and 1-aminohydantoin (AHD), respectively. The corresponding relations between nitrofurans and their metabolites, and their structures are shown in Figure 2. Furazolidone, furaltadone and nitrofurantoin are the only sources of their corresponding metabolites, indicating that we can detect the residues of AOZ, AMOZ, and AHD to reflect the amount of the parent drugs. However, the sources of SEM are diverse. There are three proven sources of SEM: first, it can be produced by the metabolism of nitrofurazone; second, it is naturally present in small amounts in some algae, shrimps and eggs; third, azodicarbonamide or hypochlorite added in flour, canned food and dairy products can be chemically changed in reactions to form SEM during processing (Hoenicke et al., 2004). Therefore, it is not rigorous to use SEM as a detection marker for nitrofurazone, but there are no better substitutes until now. Due to their potential health risks, it is not only necessary to strictly inspect the situation of nitrofuran drug residues, but also to strengthen controls at the source.

**FIGURE 3:**
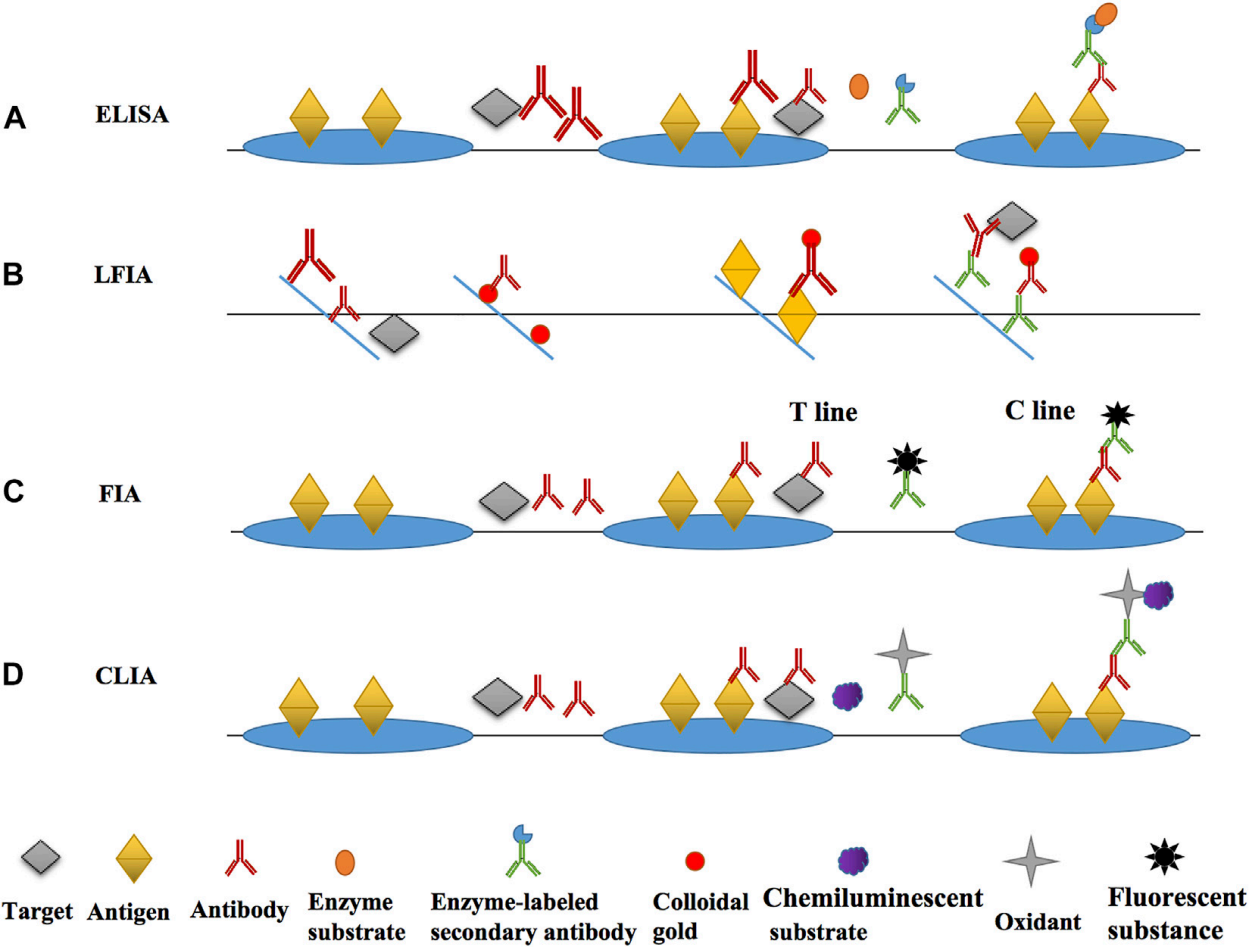
The main principle diagrams of various immunoassays. **(A)** the main principle and operation process of ELISA; **(B)** the main principle and operation process of LFIA; **(C)** the main principle and operation process of FIA; **(D)** the main principle and operation process of CLIA.

Since 1995, the European Union, the United States, China, Japan and many other countries have enacted laws and regulations to ban the use of nitrofuran antibiotics in food animals one after another (Khong et al., 2004; Chu et al., 2008). However, due to the effectiveness and low cost of nitrofurans in animal disease control, unscrupulous traders were still found using them illegally in defiance of the explicit ban. In 2003, the EU established a minimum required performance limits (MRPL) of 1 μg/kg. The MRPL value represents the highest level of drug residue that is legally tolerated in food. The European Commission will notify the exporting country and take appropriate measures if the same source or the same banned substance is found four or more times within 6 months (Vass et al., 2008). In 2015, Reference Point for Action (RPA) was established instead of the MRPL.

Currently, the RPA for nitrofuran metabolites is reduced to 0.5 μg/kg in accordance with the latest regulation 2019/1871/EC, which will take effect in 28 November 2022. According to Chinese latest limited standard GB 31650–2019, detectable AOZ, SEM, AMOZ and AHD are not permitted in chicken meat. Despite strict legislation both at home and abroad, there are still related food safety incidents occurring frequently. The regulatory system needs continuously improving, and the detection methods and technologies need constantly updating to meet higher detection requirements, especially on sensitivity and speed.

The commonly used domestic and international detection methods are the instrumental methods and the rapid immunoassay. The instrumental methods have high accuracy, but the equipment is large, expensive and complex to operate. They are suitable for the detection and verification of high requirements for accuracy and sensitivity. The existing instrumental detection methods are mainly based on chromatography (Beek and Aerts, 1985; Wang et al., 2020), chromatography-mass spectrometry (Zhang et al., 2016; Guichard et al., 2020), and fluorescent materials are combined to improve detection sensitivity (Yu et al., 2018; Luo et al., 2019).

Immunoassay and instrumental methods complement each other. Mostly, immunoassay is one of the most commonly used methods for the rapid quantitative detection (Gao et al., 2018). There are many representative products, such as early pregnancy test strips, etc. Compared to large instrumentation, the components of the immunoassay are relatively simple, requiring lower expertise and operational ability of the operator. Immunoassay have the advantage of low cost, and are popular and suitable for the screening of large quantities of samples (Vázquez et al., 2021). The characteristics of the main immunosensors we have summarized in Table 1. In this paper, we have summarized and discussed the immunological methods for the detection of nitrofuran antibiotics in animal tissue, and systematically classify the existing detection methods according to different principles and signaling molecules, expecting to promote the development of immunological detection techniques for nitrofurans.

**TABLE 1:** Comparison of various immunization methods (Yan et al., 2020a; Wang et al., 2018c; Zhang et al., 2018; Yang et al., 2012).

Detection Method	Advantage	Insufficient	External Light Source	Interpretation of Results	Detection Time	LOD	Sample Matrix	References
ELISA	Simple, high, sensitivity	Professional operation, Prone to false positives and false negatives, low repeatability	No	Enzyme labeled instrument	2.5–3 h	0.01	pork liver	Yan et al. (2020a)
						μg/L		
LFIA	Simple, fast	low sensitivity, Prone to false positives and false negatives	No	Naked eye/Reader	15–30 min	0.5–0.75 μg/L	fish, chicken	(Wang et al., 2018c)
FIA	High, Sensitivity, Reagent safety	High requirements for instruments, Unstable reagent	Yes	Fluorescence reader	15–30 min/2.5–3 h	0.021 μg/L	fish, shrimp	Zhang et al. (2018)
CLIA	high sensitivity, Wide linear range	Short luminescence process, High background value	No	Chemical luminescence immunity analyzer/Ammeter	15–30 min/2.5–3 h	0.09 μg/L	shrimp	Yang et al. (2012)

## 2 The Key Recognition Elements

### 2.1 Antigen Synthesis

The key recognition elements in biosensors are mostly antigens (Ag) and antibodies (Ab), as well as aptamers, molecularly imprinted polymers (MIP), etc. Immunoassays based on antigen-antibody specific reactions have been widely used for the detection of a wide range of compounds in the environment, food and other fields. In the immunoassay process, the most important reaction recognition element is the antibody. The commonly used antibody format is IgG, which detects the intended targets qualitatively and quantitatively by binding to a specific epitope. Usually, the higher the affinity of the antibody and the more stable it is, the higher the sensitivity of the method is (Cooper et al., 2017c). The key factor affecting the quality of the antibody is the structure of the antigen, involving the hydrophobicity (Baldofski et al., 2018), size and structure of the incomplete antigen (Cooper et al., 2017b). Therefore, the basis and key to developing highly sensitive immunoassays is to obtain antigens and antibodies with high affinity and high specificity (Dengl et al., 2016; Guillermo et al., 2019). The key to develop antibodies with desired affinity and specificity is hapten design (Shao et al., 2021). Since the molecular mass of nitrofuran metabolites is less than 1,000 Da, they are small molecule compounds that are not immunogenic and cannot easily bound to carrier protein (Cooper et al., 2017b). Therefore, when preparing antigens and assays, derivatization is usually performed first to increase the molecular mass and metabolite ionization efficiency before assaying and binding to carrier proteins. Because the derived hapten was much larger than AOZ, it possessed enhanced immunogenicity, as well as a carboxyl group that allowed it to be conjugated to a carrier protein (Cooper K. M. et al., 2017). The commonly used derivatization reagents are 4-carboxybenzaldehyde (4-CBA), 2-nitrobenzaldehyde (2-NBA), 4-nitrobenzaldehyde (4-NBA), etc. Leitner et al. (Leitner et al., 2008) compared the derivatives produced by 2-NBA and 4-NBA, and the results showed that the derivatization of 2-NBA was more effective with an efficiency greater than 70%. In the preparation of immune antigens and solid phase antigens, the commonly used carrier proteins are bovine serum protein (BSA), ovalbumin (OVA), and keyhole limpet hemocyanin (KLH). The method of coupling to the carrier protein depends on the structure of the incomplete antigen. When using CBA as a derivative, the activated ester method (Chang et al., 2008), carbodiimide method (Le and Yu, 2015; Liu et al., 2015) and mixed acid anhydride method (Cooper et al., 2004) can be used for antigen synthesis. Glutaraldehyde and diazotization methods can be used to obtain antigens when NBA derivatization is used.

### 2.2 Antibody Preparation

Obtain relevant specific antibodies by immunizing animals with antigens, its performance was characterized by enzyme-linked immunoassay (ELISA) in liquid phase system. The binding ability of antibodies to compounds can also be studied through computational chemistry analysis, which provides 3D conformational and quantitative information such as conformation, electrostatic potential, and molecular hydrophobicity (Shao et al., 2021). In 2007, Cooper et al. successfully prepared the first polyclonal antibodies against SEM and AOZ (Cooper et al., 2004; Cooper et al., 2007). In the same year, anti-AHD polyclonal antibodies were also produced by Liu et al. (Liu et al., 2007). However, polyclonal antibodies can bind to antigens with different epitopes and are prone to cross-reactivity, so they cannot meet the requirements of highly sensitive detection methods because of their low specificity. Subsequently, Gao et al. developed a monoclonal antibody (McAb) against SEM by derivatizing SEM with 4-CBA and preparing an immunogen or coating antigen in combination with bovine serum albumin (BSA) and ovalbumin (OVA), and the monoclonal antibody obtained was highly specific for CPSEM with an IC_50_ value of 1.3 μg/L (Gao et al., 2007). Monoclonal antibodies produced by a single parental cell bind only to the same epitope of the same antigen with higher specificity and sensitivity (Zhou et al., 2021), and immunoassays based on these antibodies are more sensitive, and after the cell lines are obtained, the corresponding monoclonal antibodies can be prepared indefinitely when stored properly (Li et al., 2020).

## 3 Research Progress of Immunosensors

The main immunosensor methods based on the antibody for nitrofuran detection was divided into optical and electrochemical immunosensors according to the current literatures. The optical immunosensor included ELISA, lateral flow immunochromatography (LFIA), fluorescence immunoassay (FIA), chemiluminescence immunoassay (CLIA), and surface-enhanced Raman scattering (SERS) immunoassay (Meng and Xi, 2011). A immunosensor commonly comprised of four parts, which were immunological bio-recognition element (iBRE), transducer, amplifier, and detector. The key characters of iBRE were in accordance with the recognition of antigens and antibodies. The interaction of BRE with its target generated a readable signal which converted from the transducer (Chandra, 2016).

### 3.1 Optical Immunosensor

#### 3.1.1 ELISA

ELISA was firstly developed by Engvall and Perlmann in 1971 and was the most classical label-free liquid phase immunoassay (Engvall, 1971; Aydin, 2015). According to the classification of competition principle, it can be broadly splited into two types, competitive ELISA and non-competitive ELISA (Bai et al., 2021). Small molecules, like metabolites of nitrofuran antibiotics, are often detected using competitive ELISA. For example, during the detection of Nitrofural, SEM and the encapsulated antigen compete to bind the detection antibody, and then an enzyme-labeled secondary antibody that can bind to the detection antibody and a colorless substrate which can produce coloration under enzyme catalysis are added, so that the presence of the target can be determined qualitatively and the concentration of the target can be determined quantitatively after the coloration is measured by an microplate reader (Thiha and Ibrahim, 2015). The enzymes that can be used in ELISA include horse radish peroxidase (HRP), alkaline phosphatase (ALP), glucose oxidase (GOD), etc. The enzyme and substrate often used in most studies are HRP and TMB, respectively. The colorless TMB acts as a hydrogen donor and reacts with the hydrogen peroxide catalyzed by HRP to produce blue-green oxidized quinones.

Wu et al. et al. (Wu et al., 2020) gave a detailed introduction to ELISA studies to detect AOZ, SEM, AHD, and AMOZ before 2020. Yan et al. (Yan et al., 2020b) combined Au NPs into conventional ELISA for sensitive detection of AMOZ, thereby improving detection sensitivity. In this system, a large number of reporter antibodies and horseradish peroxidase molecules are loaded onto Au NPs to amplify the colorimetric signal. Under optimized conditions, the detection limit of signal amplification ELISA reaches 10^−2^ ng/ml, and the sensitivity is 500 times higher than that of traditional ELISA. In 2020, Gaudin evaluated the performance of ELISA kits from R-Biopharm in Germany and Europroxima in the Netherlands for detecting nitrofuran metabolites in aquatic products (fish, shrimp), and the results showed that the false positive rates for both brands were below 9%, indicating the reliability and accuracy of the kit method (Gaudin et al., 2020).

#### 3.1.2 LFIA

LFIA, a type of lateral flow biosensors (LFB), was firstly developed in 1981 (Glad and Grubb, 1981) and was commercially used in pregnancy test strips. Now it has been widely used in food, medicine and other fields (Glynou et al., 2003), uch as SARS-CoV-2 (Glynou et al., 2003; Ernst et al., 2020; Yin et al., 2021a). LFIA relies on cotton thread paper or paper to display results within minutes. Probes are the most critical component of the LFIA, and they are synthesized to serve numerous purposes. Next, the LFIA will be categorized and introduced by the type of probe: gold-labeled probes, metal composite probes, organic non-metallic probes.

##### 3.1.2.1 Gold-Labeled Probes

Colloidal gold immunochromatography test is a typical, most widely used and best established LFIA methods for commercial product (Yao et al., 2016; Nardo et al., 2021). The detection probe is synthesized by combining the label with the antibody, and the probe captures the target and the antigen on the test line (T line) during the flow process on the solid phase carrier such as nitrocellulose membrane. Through the color comparison between the T line and the control line (C line) on the test paper, qualitative or semi-quantitative rapid detection is realized. Wang et al. (Wang et al., 2018b) developed a colloidal gold-based multiplex LFIA for the simultaneous detection of four nitrofuran residues as AHD, AOZ, AMOZ, and SEM. Four T line and 1 C line were present on the strip, and four different antigens were immobilized on four test lines on a nitrocellulose membrane, and goat anti-mouse IgG was immobilized on the C line as a control. The specificity of the assay was good and no cross reaction was detected among the four antibodies. In recent years, colloidal gold test strips have been relatively mature products which are widely used in many areas. With the advantage of low cost and simplicity of operator, they have become the primary choice of screening large-volume samples. Some businesses and factories often use colloidal gold test strips to check the raw materials purchased and inspect quality of goods to be shipped. However, instrumental methods are superior to rapid immunoassay in some accuracy-required assays, so improving the sensitivity of test strips is very important and has market prospect. Au NPs are the most typical and commonly used signal materials in LFIA. The sensitivity of traditional colloidal gold test strips is mainly limited by the incomplete competition between free target and immobilized antigen against gold-labeled antibodies (Lou et al., 2019). Most researchers are currently investing an enormous amount of effort and time in an attempt to improve the sensitivity of the method by finding some novel materials.

##### 3.1.2.2 Novel Nano-Labeled Probes

The probe in colloidal gold test paper is mainly synthesized by physical adsorption, the binding force is weak, the detection probe is unstable, and the sensitivity is lower. The binding stability can be improved by coupling other materials to colloidal gold. Liu et al. (Liu S. et al., 2020) used polydopamine nanospheres (PDA NPs) with covalent linkage instead of colloidal gold to prepare probes coupled with antibodies, and compared their stability with that of colloidal gold probes. The results that the amount of antibody shed on the colloidal gold probes was almost twice that of the PDA NPs probes indicated that the PDA NPs-Ab were more stable, and the detection limits (LOD) of AOZ in spiked milk powder, shrimp and pork samples were 5 μg/L, 5 μg/L and 4 μg/L, respectively. Co_3_O_4_ is a brown transition metal oxide, and Su et al. (Su et al., 2020) developed a novel LFIA biosensor using Co_3_O_4_ NPs as a signal marker instead of colloidal gold. The small size of Co_3_O_4_ enhances the sensitivity and shortens the reaction time by reducing the spatial potential resistance and increasing the flow rate. Under the optimal conditions, the detection can be completed within 6 min, with a visual detection limit (vLOD) for AOZ of 0.4 μg/L. The LOD of this method is lower than that of conventional colloidal gold test strips.

Food matrices are often complex, and excluding matrix interference is another effective mean of improving detection sensitivity when performing actual sample testing. The superparamagnetic nanoparticles have a large surface area, and the magnets enable rapid separation of the target from the impurities in solution and sample enrichment, thus increasing the reaction rate and shortening the determination time (Giakisikli and Anthemidis, 2013). Yan et al. (Yan et al., 2018) established a LFIA of detecting AOZ, based on a dual-probe magnetite nanoparticles (MNPs) labeled probe. The amplified signal benefited from high affinity between the two probes of MNPs-labeled murine monoclonal antibody (MNPs-Ab) and goat anti-mouse antibody (MNPs-GAMA), and generated a dual-probe network complex with a detection limit of 0.044 μg/L, which was 10 times more sensitive than the conventional Au NPs based LFIA, and was successfully applied to the detection of AOZ in milk. Based on the theory that streptavidin (SA) is a natural receptor for biotin and can bind to biotin (Fang et al., 2013), Wu et al. (Wu et al., 2019) combined the two signal amplification methods of biotin-streptavidin and magnetic beads, and established the method for detection of SEM, AHD, AOZ, AMOZ, the LODs were 7.20 ng/L, 11.58 ng/L, 7.24 ng/L and 2.31 ng/L, respectively. In recent years, latex microspheres have developed rapidly due to their high stability. Wang et al. (Wang J. et al., 2018) established a LFIA using red latex microspheres as tracer markers. Four different coating antigens were immobilized on nitrocellulose (NC) membranes as capture agents. Quantitative LOD (qLOD) of four main nitrofuran metabolites. The LOD of SEM, AHD, AMOZ and AOZ are 0.02–0.1 g/kg for chicken, 0.02–0.15 g/kg for fish and 0.03–0.12 g/kg for shrimp, respectively, and the recovery rate is 73.5–109.2%, the coefficient of variation is less than 15%.

##### 3.1.2.3 Metal Composite Probes

Composites are also often used to detect the sensitivity of novel nanomaterials in combination with conventional materials. Su et al. (Su et al., 2021c) designed and synthesized a snowman-like asymmetric Au-SiO_2_ Janus NP, which combined two different physicochemical properties, where the Au NPs side mainly served as a site for antibody binding and signal provision, while the SiO_2_ side mainly stablized the complex. With the unique asymmetric nanostructure, only the Au NPs side can interact with the target through specific antigen-antibody interactions, which can significantly improve competition efficiency. This method has been successfully applied to chicken, pork, honey and beef samples with vLOD of 0.8 ng/g, 0.16 ng/g, 0.4 μg/L and 0.16 μg/L, respectively. To overcome these limitations, Su et al. (Su et al., 2021b; a) used MnO_2_-Au for dual-signal immunoassay. On the one hand, vLOD is performed through color changes, and on the other hand, quantitative detection is achieved by using thermal infrared imager to record and convert conventional detection signals into thermal signals. The vLOD of this method for AOZ is 1 μg/L, and the qLOD is 0.43 μg/L, and the recovery rate is 80.6–106%. Yin et al. (Yin et al., 2021a) proposed an immunochromatographic method based on an immune scaffold to simultaneously detect nitrofurazone and furazolidone. The immune scaffold is composed of labeled anti-antibody IgG (Ab_2_) and two anti-analyte monoclonal antibodies (Ab_1_). Using MIL-101(Cr)@AuNPs nanocomposite as the signal label, the amount of Ab_1_ can be applied 6 times less than the conventional method, and the signal intensity is similar. This method greatly reduces the cost.

##### 3.1.2.4 Organic Material Probes

The utilization of polymer/metal organic framework (MOF) nanocomposites in various biomedical applications has been widely studied due to their unique properties that arise from MOFs or hybrid composite systems (Giliopoulos et al., 2020). Yin et al. (Yin et al., 2021b) reported a nano-signal labeling strategy in which amino-terminated zirconium MOFs (NU66) were used to construct the matrix material, and Au NPs were directly fixed on the surface of NU66 as a connector between metal organic MOFs and antibodies, and gave NU66 excellent Biocompatibility and bright color signal marking. The vLOD of this method for 3-CPAOZ is 0.6 ng/ml. The existing qualitative and quantitative detection immunoassay techniques are limited in their application and development due to their poor sensitivity and the need for cumbersome quantitative equipment.

#### 3.1.3 FIA

The FIA is mainly about using a nanomaterial, like a fluorescent substance, instead of colloidal gold or enzyme labelled antibody to detect and quantify the content of the target based on the fluorescence intensity, thus improving the detection sensitivity. According to different reaction systems and the specific fluorescent substances, these methods can be classified into several types.

Fluorescent substances mainly include substances with fluorescence effect, such as fluorescein and its derivatives (Hu et al., 2016), rhodamine (Feng et al., 2002), quantum dots (QDs) (Zhou et al., 2020), lanthanide chelates (Lee et al., 2020), essence dyes (Liu J. et al., 2020) and phycoerythrin (Yang et al., 2020), as well as some substances that can emit fluorescence under the action of enzymes, which usually produce visible light after the irradiation of ultraviolet or X-ray. However, the sensitivity of the traditional fluorescein-labeled (fluorescein, etc.) immunoassay is greatly limited by factors such as large background interference, small Stokes shift (Yuan and Wang, 2005), and disorganized background scattered light.

QDs have enriched fluorescence properties which includes broad excitation spectrum, narrow emission spectrum and photostability. At the same time, there are discrete capping agents and different functional groups on its surface, thus increasing the sensitivity of the method (Biranje et al., 2021). Xie et al. (Xie et al., 2021) established both ic-ELISA and Fluorescence-Linked Immunosorbent Assay (FLISA) based on QDs to detect AMOZ. The IC_50_ values of ic-ELISA and FLISA were 0.11 and 0.09 μg/L, respectively, the recovery rate is 81.1–105.3%, and the coefficient of variation was 4.7–9.8%. Both methods are suitable for effective high-throughput detection methods, and in contrast, FIA has higher sensitivity. Carbon dots (CDs) are a class of zero-dimensional fluorescent nanomaterials based on carbon, and have emerged in recent years (Raveendran and Kizhakayil, 2021). Compared with traditional fluorescent nanomaterials (e.g., quantum dots), it can synthesize CDs in a simple way, and have the advantage of low cost, good photostability and low toxicity. However, bare CDs are prone to aggregation quenching during labeling, while presilylated CDs can effectively alleviate this phenomenon, but additional surface modifications are still required to label antibody probes. Dong et al. have prepared carbon-dot fluorescent immunoprobes for detection of chloramphenicol (Dong et al., 2020). Up to now, in the FIA of nitrofurans, there are no studies on the use of CDs-binding antibodies or antigen preparation Immune probes. The application of carbon dots in FIA for the detection of nitrofurans is worth exploring in the future. The lanthanide chelates, as represented by Eu^3+^, have a wide range of excitation wavelength and a narrow range of emission wavelength, which were suitable for time-resolved FIA (TR-FIA), eliminating the interference factors of common fluorescence methods and improving the sensitivity. The TR-FIA was first developed by Kakabakos and Kosravi for detecting progesterone in serum in combination with the biotin-streptavidin system (Kakabakos and Khosravi, 1992). Zhang et al. (Zhang et al., 2018) established an immunoassay method that combines the TR-FIA with the biotin-streptavidin amplification system, which can sensitively detect the residual AMOZ in aquatic tissues. This method shows high sensitivity and specifications for 2-NPAMOZ, with IC_50_ of 0.190 μg/L, LOD of 0.019 μg/L, and detection range of 0.025–10 μg/L. The LOD in fish and shrimp was 0.021 μg/kg, the recovery rates were 84.1–107.0% and 80.9–98.4%, respectively, and the average RSD was less than 10%. Cai et al. (Cai et al., 2021) prepared copper nanoclusters (Cu NCs) as fluorescent probes using glutathione (GSH) as a protective agent and ascorbic acid as a reducing agent. Cu NCs show blue fluorescence with a peak concentrated at 426 nm, and have excellent water solubility, stability and dispersibility. Based on the inner filtration effect and static quenching mechanism, Cu NCs were used to detect furazolidone in bovine serum samples. Under optimal detection conditions, the LOD was 0.012 μM. Wang al (Wang et al., 2022). synthesized a composite fluorescent probe based on Ag_2_S QDs/gC_3_N_4_ for use in detect NFZ. TR-FIA spectroscopy and UV-visible absorption spectroscopy results indicate that the possible detection mechanism of Ag_2_S QDs/gC_3_N_4_ to NFZ is proposed as the internal filtering effect (IFE). The possible interaction between Ag_2_S QDs/gC_3_N_4_ and NFZ was revealed by Multiwfn wavefunction analysis, and the mechanism of fluorescence detection was further revealed from the atomic scale. Combining experiments and theoretical calculations, a sensing mechanism for the formation of hydrogen-bonded non-fluorescent ground-state complexes is proposed. The results showed that the linear detection range of Ag_2_S QDs/gC_3_N_4_ for NFZ was 0–30 μM, and the low LOD was 0.054 μM. This work demonstrates that the Ag_2_S QDs/gC_3_N_4_ composite has the ability to detect NFZs with high efficiency and sensitivity.

#### 3.1.4 CLIA

CLIA (Huang et al., 2018) is a phenomenon in which some substances (luminol etc.) absorb energy, and then produce electron leaps during a chemical reaction, thus releasing sufficient energy to emit light. It can be divided into three types: direct chemiluminescence immunoassay, enzymatic chemiluminescence immunoassayand electrochemiluminescence immunoassay.

##### 3.1.4.1 Direct CLIA

In the direct CLIA, antibodies or antigens are directly labelled by luminescent agents, such as acridinester compounds (Khan et al., 2014), which can emit light by oxidant action or enzyme catalysis, and the content of target can be quantified by measuring light signals (Fan et al., 2009).

Recently, Xu et al. (Xu et al., 2021) found that, in addition to traditional luminescent agents, CPAOZ McAb can be specifically and directly stained by CBB-R in Coomassie Brilliant Blue (CBB), a chemical dye with a concentration of 0.05%, and be applied to the LFIA platform for detection of FZD. The results showed that the actual samples, such as honey, chicken intestine and shrimp, were detected with high sensitivity (LOD was 2 μg/kg) and high specificity. As a noble method without nanoparticle labeling, it is rapid, low cost, easy to operate be commercialized. Liang et al. (Liang et al., 2019) developed a magnetic powder chemiluminescence kit. Using magnetic particle chemiluminescence enzyme-linked immunoassay (MCLIA) combined with full enzyme-linked immunoassay, simultaneous quantitative detection of SEM, AOZ, AMOZ and AHD. It was used to fish, pork, chicken and shellfish samples, and the compliance rate with the national standard method is more than 98%. Liu et al. (Liu et al., 2013) developed an CELISA combined with chemiluminescence to study and optimize the effects of the substrate luminol, iodophenol and carbamide peroxide on the performance of the assay. The IC_50_ value of this method was 0.14 μg/L, the linear working range is between 0.03 and 64 μg/L, and the LOD was 0.01 μg/L. The recovery rates of four fish, shrimp, honey and egg samples with different concentrations of NPAMOZ 92.1–107.7%.

##### 3.1.4.2 Enzymatic CLIA

Enzymatic CLIA often uses HRP or ALP to act on luminol and 1, 2-dioxane derivatives (AMPPD) to emit light, so as to quantify the content of the target analyte by measuring light signals. The principle of this method is the same as ELISA, except that luminol is used as the substrate (Fan et al., 2009).

Liu et al. (Liu et al., 2012) developed a highly sensitive chemiluminescence method for determination of nitrofurans (NFs) based on nanosilver (Ag NPs) as an enhancer. Ag NPs can catalyze the production of OH center dot radicals and enhance the chemiluminescence intensity of the luminol-H_2_O_2_-NFs system.

#### 3.1.5 SERS-Based Immunoassay

Li et al. developed a new ultra-sensitive competitive LFIA based on SERS for the direct detection of AMOZ in tissue and urine samples. The AMOZ detection was completed by measuring the specific Raman scattering intensity of the MBA on the test line of the LFIA test strip. The determination can be completed within 15 min, and the IC_50_ value and LOD determined by AMOZ are 40 ng/L and 0.28 ng/L, respectively.

In addition to incorporating nanomaterials as signal markers, there are also nanomaterial-free labelling methods that use some staining materials as direct signal molecules. Dou et al. (Dou et al., 2019) developed a LFIA based on non-nanomaterials, that is, LFIA based on crystal violet (CV) dyeing. The vLOD and qLOD of FZD were 7 μg/L and 1.53 μg/L, respectively. This nanomaterial-free technology overcomes the inherent limitations of nanomaterials. Zhang et al. (Zhang et al., 2020) developed an efficient geomagnetic solid-phase extraction (MSPE) surface-enhanced Raman scattering (SERS) method for the detection of aromatic amines and nitrofurans in real samples using CoFe_2_O_4_@HNTs/AuNPs substrates. The CoFe_2_O_4_@HNTs/AuNPs substrate exhibited excellent SERS activity (high sensitivity, good reproducibility and reproducibility), pH stability (3.0–11.0), and good MSPE ability (rapid speed within 5 min) Magnetic enrichment/separation capability) Magnetic beads filled with CoFe_2_O_4_ inside halloysite nanotubes (HNTs) can avoid particle aggregation, endow the substrate with rapid magnetic separation capability, simplify pretreatment procedures, and reduce complex matrix interference. The surface AuNPs can generate electromagnetic enhancements and hot spots to amplify the Raman signals of target molecules enriched/condensed by HNTs. Nitrofurantoin was determined with a linear range of 0.05–1.0 mg/L and a LOD as low as 0.014 mg/L. Bi et al. (Bi et al., 2022) prepared β-cyclodextrin (β-CD) protected AuNPs/γ-Al_2_O_3_ nanoparticles as substrates and developed a novel SERS method for the detection of nitrofurazone. Optimal experimental conditions were obtained by one-factor procedures and response surface modeling. A linear relationship between the SERS intensity and the concentration of nitrofurazone was established in the range of 3.3–667.0 nmol/L (I_SERS_ = 508.96c + 31,987.87, c: nmol L^−1^, *R* ^2^ = 0.996).

### 3.2 Electrochemical Immunosensor

Electrochemical immunosensor combines electrochemistry and immunoassay by detecting the relative change of electrical signals before and after the binding of the probe to the antigen to quantify the amount of the target. In electrochemical immunoassay, carbon nanotubes (CNT), carbon nanoparticles (CNP), nanodiamond-graphite (NDG), graphene oxide (GO) and other carbon nanomaterials are often used to modify the surface of glassy carbon electrodes for higher detection sensitivity (Shahrokhian et al., 2016). Compared with conventional electrodes, thin-film gold electrodes modified with precious metals are more sensitive and can be mass-produced. He et al. (He and Liu., 2019) developed an electrochemical sensor based on Au/graphene-modified thin-film gold electrode. The introduction of gold nanoparticles onto the graphene modified thin film gold electrode can effectively enhance the electron transfer. Under the optimal conditions, the sensor detected SEM with a LOD of 0.0047 μmol/L and was successfully applied to pork with a 40-fold increase in sensitivity compared with the electrode modified with CMWCNTs. Wang et al. (Wang et al., 2021) developed a new competitive electrochemical immunosensor based on square wave voltammetry (SWV) response to quantitatively detect AHD. Au nanoparticles are grown on the surface of Ce-based metal organic framework (Ce-MOF) as the signal amplification platform of the sensor. Methylene blue (MB) loaded Au at Pt and coated antigen (OVA-AHD) were connected as signal markers. The target competes with the coating antigen for the antibody, resulting in a decrease in the number of signal probes bound to the antibody. The concentration of AHD can be determined by SWV detection of the MB signal loaded on the signal label. Under optimal conditions, the LOD was 1.35 × 10^−7^ μg/L.

Niu et al.(Niu et al., 2021)constructed a bimetallic Co./Ni-MOF-derived hollow NiCo_2_O_4_@C composite modified glassy carbon electrode (NiCo_2_O_4_@C/GCE) and applied it to the simultaneous detection of FZD and Chloramphenicol (CAP). NiCo_2_O_4_@C/GCE exhibits excellent electrocatalytic performance for simultaneous determination of FZD and CAP. NiCo_2_O_4_@C/GCE has a linear range of 0.5–240 µM for FZD and 0.5–320 µM for CAP, with a lower detection limit of 8.47 nM for FZD and 35 nM for CAP. Mechanistic studies show that the reduction of FZD and CAP on NiCo_2_O_4_@C/GCE are both four-electron and four-proton processes. In addition, the sensor achieved ideal recoveries for simultaneous determination of FZD (95.85–103.9%) and CAP (95.72–104.4%) in milk and honey by standard addition method. Maheshwaran al (Maheshwaran et al., 2022). Using hydrothermally synthesized BiVO_4_@MoS_2_ hierarchical nanoheterojunction composites to develop an electrochemical sensor for FZE detection by modifying glassy carbon electrode (GCE) as a novel electrocatalyst. The electrochemical performance of 1D-2D BiVO_4_@MoS_2_ was examined by cyclic voltammetry and differential pulse voltammetry (DPV) analysis. The BiVO_4_@MoS_2_ composite exhibits excellent electrocatalytic activity for FZE sensing with linear detection ranges of 0.01–14 and 14–614 μM. The LOD of the BiVO_4_@MoS_2_ based sensor was determined to be 2.9 nM, which was far superior to other reported FZE sensors. Considerable recoveries when tested in human urine and serum samples. Cai et al. (Cai et al., 2022) combined functionalized polyoxometalates with graphene-modified electrodes through layer-by-layer assembly to achieve sensitive detection of NFZ with a LOD as low as 0.08952 μM. Direct low-level detection through real samples was achieved by accelerating its electron transfer modified electrode through [Ru-PMo_12_/PDDA-GO]_3_.

### 3.3 Others

In addition to the above mentioned methods, there are also other immunological technologies against nitrofurans residues. Liu et al. (Liu et al., 2015) developed a protein microarray method to detect AOZ in synaptic eels, and the results were similar to those of commercially available kits, with the advantages of instrument portability and visualization of the results. In response to the miniaturization, integration and intelligence trends of modern technology, microfluidic chip-based immunoassay technology has also been gradually applied to the detection of nitrofurans.

## 4 Outlook

Nitrofuran veterinary drugs, as prohibited drugs, can still be constantly detected in animal food in international and domestic trade, indicating that there is still a large technical barrier. On one hand, national regulatory agencies need to continuously amend the bill to improve and perfect the legal system with strict limit standards and requirements to control; on the other hand, it is necessary to vigorously develop and improve detection technology. The current internationally recognized method for accurate quantification is the high-precision instrumentation method. As another mainstream technology that goes hand in hand, rapid detection technology is the preferred technology for high-throughput screening, and has great potential for development and research, providing new technologies to address the problem of multiple risks of food safety coexisting. Achieving the same detection limits as RPA is not enough, the experts should strive to raise the technical level in order to detect trace levels. Efforts should be made to explore further in the direction of sensitization and micro materials, so that the detection instruments and technologies will have higher sensitivity, miniaturization and portability. The new nanomaterials should have high sensitivity, easy preparation, convenient operation, obvious color, low cost, small size, and be combined with microchips to make qualitative improvements on the rapid detection products in operability, sensitivity and convenience. Moreover, some materials with high specificity for target should be explored, such as CBB-R, which is specifically applied to the detection of nitrofurans, and many new technologies based on them will reduce the multiple risks of food safety. A wide variety of detection methods and micro instruments have blossomed, facilitating people’s daily lives and self-testing and creating great economic benefits in deed.
